# Uremic Toxins and Clinical Outcomes: The Impact of Kidney Transplantation

**DOI:** 10.3390/toxins10060229

**Published:** 2018-06-05

**Authors:** Sophie Liabeuf, Lynda Cheddani, Ziad A. Massy

**Affiliations:** 1 Division of Clinical Pharmacology and Clinical Research Department, Amiens University Medical Center, 80054 Amiens, France; 2 EA 7517, MP3CV laboratory, CURS, Jules Verne University of Picardie, 80054 Amiens, France; 3 Division of Nephrology, Ambroise Paré University Medical Center, APHP, Boulogne Billancourt, 92100 Paris, France; lynda.cheddani@aphp.fr (L.C.); ziad.massy@aphp.fr (Z.A.M.); 4 INSERM U1018, Team 5, CESP (Centre de Recherche en Épidémiologie et Santé des Populations), Paris-Saclay University and Paris Ouest-Versailles-Saint-Quentin-en-Yvelines University (UVSQ), 94800 Villejuif, France

**Keywords:** uremic toxins, chronic kidney disease, renal transplantation, outcomes

## Abstract

Non-transplanted and transplanted patients with chronic kidney disease (CKD) differ in terms of mortality and the risk of clinical events. This difference is probably due to the difference of both traditional and non-traditional risk factors. Uremic retention solutes may constitute important non-traditional risk factors in this population. In the present review, we selected a set of uremic toxins that have been associated with harmful effects, and are an appealing target for adjuvant therapy in CKD. For each toxin reviewed here, relevant studies were selected and the relationship with hard clinical outcomes of uremic toxins were compared between non-transplanted CKD patients and transplanted patients taking into account the level of glomerular filtration rate in these two situations.

## 1. Introduction

The burden of chronic kidney disease (CKD) is substantial. According to the World Health Organization’s Global Health Estimates, around 1.5% of deaths worldwide in 2012 were attributable to CKD [1]. The complications of CKD include anemia, bone disease, and an increased risk of cardiovascular disease and cancer [2]. In patients with end-stage renal disease (ESRD), successful kidney transplantation improves quality of life and prolongs survival, relative to long-term dialysis treatment [3]. However, renal function does not usually recover completely after kidney transplantation; transplant recipients therefore constitute a particular subset of patients with CKD, most of whom have stage 2 or 3 CKD (according to the National Kidney Foundation’s classification) [4]. It is universally accepted that morbidity and mortality rates are higher in patients with altered renal function (whether transplanted or not) than in populations with normal renal function.

Indeed, recent data from the ERA-EDTA registry confirmed a substantial difference in the expected remaining lifetime between the general population and patients on dialysis. Patients on dialysis aged 20–44 are expected to live for only one-third of the estimated remaining lifetime of an age-matched general population sample—about 35 years less. As mentioned above, kidney transplant recipients fare better than those receiving dialysis. However, the life expectancy of transplant recipients aged 20–44  is still approximately 17 years shorter than that of the age-matched general population [5].

Due to the low life expectancy and high prevalence of comorbidities in patients with CKD, research in this field is focusing on the identification of modifiable risk factors. Uremic retention solutes may constitute important non-traditional risk factors in this population. The members of this large group of solutes (referred to as “uremic toxins” when they perturb normal biological functions) differ in their water solubility, protein-binding capacity, molecular weight, pattern of removal by dialysis, biological properties, and ability to produce clinical symptoms. Three subgroups of uremic toxins have been suggested: small molecules (including urea and phosphate), middle molecules (including fibroblast growth factor 23 [FGF23]), and protein-bound uremic toxins (including p-cresylsulfate [PCS] and indoxylsulfate [IS]) [6]. A substantial number of publications have reported on the circulating concentrations of individual uremic toxins in patients with CKD. Furthermore, some observational studies (but not others) have linked these uremic toxins to clinical outcomes.

For the purposes of the present review, we selected uremic toxins that are representatives for each of the three uremic toxin classes reported by European Uremic Toxin Workgroup (EUTox). The vascular and renal toxicity of these selected co-metabolites have been demonstrated extensively in experimental and clinical studies including kidney transplantation. Moreover, these co-metabolites are an appealing target for adjuvant therapy in CKD. We then compared the published data between transplanted recipients and non-transplanted patients (Table 1).

## 2. Phosphate

### 2.1. Serum Phosphate Levels

#### 2.1.1. Chronic Kidney Disease and Dialyzed Patients

It is challenging to accurately describe renal phosphate homeostasis in CKD patients [7]. Serum phosphate levels are normal in the vast majority of patients with early- or intermediate-stage CKD. Close to 90% of patients with stage 3 or 4 CKD have normal serum phosphate levels [8,9]. This degree of homeostasis is related to a reduction in tubular resorption of phosphate following a compensatory increase in the secretion of the phosphaturic hormone FGF23 and parathyroid hormone (PTH) [8,9,10,11,12]. Clinical studies have evidenced abnormally high fractional phosphorus excretion in patients with early-stage CKD [8,10,13]. In patients with late-stage CKD, these compensatory mechanisms are overwhelmed by a progressive reduction in the number of functional nephrons [11]. Serum phosphate levels then increase as a result of a positive phosphate balance, which usually leads to hyperphosphatemia in patients with ESRD [11]. In a study of 3879 patients with CKD from the Chronic Renal Insufficiency Cohort (CRIC), Isakova et al. observed that (i) the level of serum FGF23 rose as the estimated glomerular filtration rate (eGFR) declined, and (ii) rises in serum FGF23 and PTH levels preceded hyperphosphatemia; similar results have also been described elsewhere [10,11,12,14].

#### 2.1.2. Renal Transplant Recipients

Hypophosphatemia is especially common after successful kidney transplantation, and affects up to 90% of patients in the immediate post-transplantation period [15,16]. It has been suggested that the persistence of high serum levels of the phosphaturic hormone FGF23 has an important role in the pathogenesis of post-transplantation hypophosphatemia [17,18]. Most studies have described a post-transplantation decrease in serum phosphorus levels (with a return to normal values within 12 months) [19] and a concurrent decline in FGF23 and PTH levels [20] during the so-called “recovery phase”. There are conflicting reports on long-term changes in renal phosphate wasting as some researchers have found that this condition persists after the recovery phase; the induced skeletal phosphate mobilization contributes to a progressive decline in bone mineral density and an increased risk of bone fracture in kidney transplant recipients [21,22].

### 2.2. Phosphatemia and Outcomes

#### 2.2.1. Chronic Kidney Disease and Dialyzed Patients

a. Serum phosphate levels and mortality

Hyperphosphatemia is currently considered to be a risk factor for both all-cause and cardiovascular mortality in patients with CKD [9,11]. Abnormally high serum phosphate levels have been linked to an elevated risk of mortality in patients with CKD before transplantation [23,24]. In 2015, Da et al. published the results of a meta-analysis of 12 cohort studies and a total of 25,546 patients with CKD. The researchers found that an elevated serum phosphate level was an independent risk factor for kidney disease progression and mortality among non-dialysis-dependent patients with CKD [25]. In two other meta-analyses of patients with CKD, the risk of death increased by 18% or 20% for each 1 mg/dL increment in serum phosphate [25,26]. More recently, Hou et al.’s meta-analysis of nine cohort studies (including a total of 1,992,869 patients with ESRD requiring dialysis) revealed that both abnormally high and abnormally low serum phosphate levels were independently associated with an increased risk of all-cause mortality in this population (hazard ratio (HR) [95% confidence interval (CI)] = 1.39 [1.31–1.47] and 1.16 [1.06–1.28], respectively). Moreover, serum phosphate levels were non-linearly associated with all-cause mortality in dialysis-dependent patients with ESRD [27].

Although a high serum phosphate level is undoubtedly a risk factor for mortality, it is not clear whether reducing serum phosphate levels would improve clinical outcomes in individual patients [9].

b. Serum phosphate levels and cardiovascular events

Although most of the observational studies of serum phosphate levels in patients with CKD [25,26,28,29,30,31,32,33,34,35,36,37,38,39,40,41,42] have found an independent association between hyperphosphatemia on one hand and increased mortality and cardiovascular event risks on the other, the underlying mechanisms have yet to be understood. However, the role of hyperphosphatemia has been studied for several specific outcomes, such as vascular calcification, left ventricular hypertrophy (LVH), and the progression of kidney failure [9,37,39,41,42]—all of which are well-known biomarkers for mortality in patients with CKD.

Vascular calcification is considered to be one of the mechanistic links between hyperphosphatemia and the elevated risk of cardiovascular mortality observed in patients with CKD. Data from animal studies have revealed that (i) exposure to high phosphate concentrations can upregulate osteoblast factors in vascular smooth muscle cells and may therefore be involved in vascular calcification [43,44,45,46,47,48], and (ii) this process begins in the early stages of CKD [43,49,50,51,52,53]. Moreover, clinical studies of pre-dialysis patients showed that higher serum phosphate levels were linked to arterial calcification [54] and arterial stiffness [55]. Several studies have found that the high frequency of severe calcification in patients with CKD on dialysis [49] is associated with mortality [56,57,58]. Furthermore, arterial stiffness (a putative indirect maker of vascular calcification) is reportedly an independent predictive factor for coronary heart disease, fatal stroke, and total and cardiovascular mortality in ESRD populations [59,60,61,62].

Moreover, LVH is an independent risk factor for heart failure and mortality in many populations, including pre-dialysis patients with CKD [63]. Hyperphosphatemia in patients with CKD has already been linked to LVH [64,65]. Ayus et al. subsequently reported that a decrease in serum phosphate levels was associated with a decrease in LVH. They suggested that a vascular-calcification-independent pathway might mediate the development of LVH in dialyzed patients [66,67]. To note other factors such as volume overload and hypertension could be implicate in the development on LVH. Furthermore, the results of epidemiological studies indicate that the prevalence of LVH increases as the eGFR declines (such that up to 75% of incident dialysis patients are affected), and that heart failure is a leading cause of cardiovascular death among patients with CKD or ESRD [50,68,69,70].

#### 2.2.2. Kidney Transplant Recipients

a. Serum phosphate levels and mortality

As seen for patients with CKD prior to kidney transplantation, most (but not all [71]) observational studies have found that hyperphosphataemia is independently associated with an increased risk of death in kidney transplant recipients [72,73,74,75]. Recently, Merhi et al. published the results of an ancillary study of the Folic Acid for Vascular Outcome Reduction in Transplantation (FAVORIT) trial cohort, where the serum phosphorus level was strongly associated with all-cause mortality in kidney transplant recipients; after adjustment for traditional cardiovascular risk factors, the transplant type (i.e., live vs. cadaveric donor) and the transplant vintage, each 1 mg/dL increment in serum phosphorus level was associated with a 34% increase in the mortality risk [75]. Wolf et al. [76] previously reported similar results in an analysis of a single-center cohort: each 0.9 mg/dL increment in serum phosphorus level was associated with an increase in the frequency of the composite endpoint (death or transplant failure: HR [95% CI] = 1.23 [1.08–1.40]). The association between serum phosphate levels and all-cause mortality was also evaluated by Pihlstrøm et al. among kidney transplant recipients participating in the Assessment of Lescol in Renal Transplantation trial. Serum phosphorus levels were significantly associated with all-cause mortality in unadjusted models (HR [95% CI] per 1 mg/dL increment in serum phosphorus: 1.23 [1.07–1.43]), but not in models adjusted for other mortality risk factors (HR [95% CI] = 1.07 [0.89–1.28]) [77].

b. Serum phosphate levels and cardiovascular events

Merhi et al. [75] reported that in the FAVORIT trial cohort, each 1 mg/dL increment in serum phosphorus level was associated with a 14% increase in the risk of cardiovascular disease. In order to identify covariables that associated with rapid cardiovascular risk progression, Kerry et al. studied major adverse cardiac events in clinically stable kidney transplant recipients. They found that even upper-normal serum phosphate levels were predictive of worse cardiovascular risk scores in clinically stable kidney transplant recipients [78].

Recently, Von Londen et al. reported that post-transplant hypophosphatemia in a cohort of 957 kidney transplant recipients was associated with a lower risk of cardiovascular (but not all-cause) mortality. The researchers suggested that the effects of this association were exerted through FGF23. As mentioned above, post-transplant renal phosphate wasting leads to a decline in circulating levels of FGF23 and PTH. A more efficient reduction in FGF23 levels may indeed contribute to the lower risk of cardiovascular mortality in kidney transplant recipients who develop hypophosphatemia within 12 months, since FGF23 has been implicated in cardiovascular disease [79,80,81] and a higher risk of cardiovascular mortality in kidney transplant recipients [76,82].

The development of hypophosphatemia is associated with cardiovascular mortality (fully adjusted HR [95% CI] = 0.37 [0.22‒0.62]), but not noncardiovascular mortality (fully adjusted HR [95% CI] = 1.33 [0.9‒1.96]), or all-cause mortality (fully adjusted HR [95% CI] = 1.15 [0.81‒1.61]) [83].

## 3. Trimethylamine-N-Oxide (TMAO)

### 3.1. Serum TMAO Levels

The metabolite TMAO is produced from trimethylamine-containing nutrients (such as carnitine, phosphatidylcholine, and choline), which are abundant in the Western diet. Trimethylamine-N-oxide promotes vascular inflammation, modulates platelet function, and prompts the generation of a prothrombotic phenotype in vivo; it seems to be a mediator of cardiovascular disease [84].

#### 3.1.1. Chronic Kidney Disease and Dialyzed Patients

In a study of patients with heart failure, it was noted that there was an inverse correlation between TMAO levels and eGFR (*r* = −0.55; *p* < 0.001) [84]. Indeed, serum TMAO concentrations increase substantially as kidney function worsens. In a cohort of patients with CKD, serum TMAO concentrations displayed a strong, inverse association with eGFR (*r*^2^ = 0.31, *p* < 0.001). The (median (interquartile range) serum TMAO concentration was markedly higher in patients on dialysis (94.4 μM (54.8‒133.0 μM)) than in healthy controls (3.3 μM (3.1–6.0 μM; *p* < 0.001)) [85].

In another study, the mean ± standard deviation (SD) TMAO concentration in pre-dialysis plasma (99.9 ± 31.9 μM) was significantly (*p* < 0.05) higher in patients on dialysis than in healthy subjects (37.8 ± 20.4 μM). The TMAO still accumulated between treatments but was efficiently removed by a single hemodialysis session [86].

#### 3.1.2. Kidney Transplant Recipients

To investigate whether the extreme elevations in serum TMAO observed in advanced CKD are reversed when kidney function is restored, Stubbs et al. [85] evaluated this parameter before and three months after successful kidney transplantation. All transplant recipients demonstrated a marked reduction in serum TMAO levels; the median TMAO concentration was 71.3 mM before transplantation and 11.4 mM afterwards (*p* = 0.03). Similarly, Missailidis et al. [87] recently reported that serum TMAO levels decline rapidly after kidney transplantation and remain low for at least the following two years.

In a recent prospective study, the levels of various uremic toxins (including TMAO) were monitored in 51 kidney transplant recipients at the time of transplantation and then 7 days, 3 months, and 12 months later. Levels of all the analyzed uremic toxins fell rapidly in the first seven days post-transplantation. When compared with 51 control patients with CKD (matched for age, gender, and presence of diabetes mellitus and eGFR), the serum TMAO level was significantly lower on day 7 (*p* = 0.007), but not at month 3 (*p* = 0.50), or month 12 (*p* = 0.87) [88].

### 3.2. TMAO and Outcomes

Animal studies have suggested that the intestinal microbiota have a role in the pathogenesis of atherosclerosis in patients with a phosphatidylcholine-rich diet via the formation of the metabolite trimethylamine and its conversion to TMAO [89].

#### 3.2.1. Chronic Kidney Disease and Dialyzed Patients

The robustness of TMAO as a predictor of cardiovascular risk was subsequently demonstrated in large cohorts of non-CKD patients [90]. In a study of 4007 patients undergoing elective coronary angiography, elevated plasma TMAO levels were associated with an increased risk of a major adverse cardiovascular event (HR [95% CI] for highest vs. lowest TMAO quartile = 2.54 [1.96‒3.28; *p* < 0.001]) [89].

The relationship between high levels of TMAO and cardiovascular events in patients with a high cardiovascular risk has been extended to patients with CKD. Indeed, Tang et al. examined the relationship between the fasting plasma TMAO level and all-cause mortality over a 5-year follow-up period in 521 subjects with stable CKD (eGFR < 60 mL/min per 1.73 m^2^). After adjusting for traditional risk factors, the researchers found that high-sensitivity C-reactive protein, estimated GFR, and elevated serum TMAO levels were predictive of 5-year mortality risk (HR [95% CI] = 1.93 [1.13‒3.29]; *p* < 0.05) [91]. Similarly, in a cohort of patients with CKD, the serum TMAO level was an independent predictor of the coronary atherosclerosis burden and long-term mortality, independently of traditional cardiac risk factors (HR [95% CI] = 1.26 [1.13‒1.40] per 10 μM increment in the TMAO concentration; *p* < 0.001) [85].

#### 3.2.2. Kidney Transplant Recipients

There are few published data on the association between TMAO levels and hard outcomes in kidney transplant recipients. Some studies of patients with CKD have shown that high levels of TMAO predicted reduced 5-year survival after multivariate adjustment, although few transplanted patients were assessed [85,87]. Kidney transplant recipients have not been specifically studied in this field.

## 4. FGF23

### 4.1. Serum FGF23 Levels

#### 4.1.1. Chronic Kidney Disease and Dialyzed Patients

Many studies have focused on the metabolism of FGF23, which is greatly modified by CKD [92]. In adult patients with CKD, circulating FGF23 levels increase as the GFR falls [10], and this rise occurs from early-stage CKD onwards. The accumulation of FGF23 is at least partly due to decreased renal clearance, although the results of several studies suggest that FGF23 production also increases [93,94]. The elevation of FGF23 stimulates the excretion of the excess serum phosphate [92,95], and it contributes to a decrease in 1,25-dihydroxyvitamin D synthesis [14]. Thus, serum FGF23 levels increase in early-stage CKD (before serum phosphate levels becomes abnormal) [92,95], maintains a neutral phosphate balance, has a reduced renal calcitriol production, and thereby stimulates PTH secretion [96]. In a study of 3879 patients with CKD from the CRIC, Isakova et al. concluded that the serum FGF23 and PTH levels increase as eGFR falls, before the onset of hyperphosphatemia [10,11,12,14].

#### 4.1.2. Kidney Transplant Recipients

In the immediate aftermath of successful kidney transplantation, FGF23 levels remain high. The failure of osteocytes to reduce FGF23 secretion immediately upon the recovery of renal function may be due (at least in part) to their prolonged stimulation during the months preceding transplantation [97]. Patients with elevated post-transplantation FGF23 levels might be those with the highest pre-transplantation levels [18]. During the recovery period following successful kidney transplantation, FGF23 levels decline drastically but can remain higher than in GFR-matched, nontransplanted patients with CKD [94,98]. It is still not clear why excessive FGF23 secretion persists after transplantation or how this excessive secretion eventually resolves [17,99]. Bhan et al. [17] hypothesized that the prolonged, excessive bone stimulation during the months preceding kidney transplantation induces resistance to stimuli that would normally inhibit FGF23 secretion.

Evenepoel et al. [18] performed a single-center, prospective, observational study that included 41 kidney transplant recipients. One of the objectives was to describe the natural history of FGF23 in the early post-transplantation period. The mean serum FGF23 level had fallen by 95% at month three after a successful kidney transplantation, although levels remained elevated in most patients. The pre-transplant FGF23 level was the strongest predictor of the post-transplant FGF23 level. In this study, there was no association between serum FGF23 and serum phosphate in the early post-transplantation period. Despite lower serum phosphate levels, serum FGF23 levels at month three were substantially higher than those previously observed in non-transplanted patients at a similar CKD stage [14]. Evenepoel et al. suggested the existence of “tertiary hyperphosphatoninism”, in which FGF23 secretion continues to be elevated. As suggested previously by Bhan et al. [17], they also supposed that the endocrine osteoblast cells may have developed resistance to the stimuli that normally inhibit FGF23 secretion during uremia, as a result of the preceding months or years of excessive FGF23 stimulation. The pathophysiological mechanisms underlying this “tertiary hyperphosphatoninism” have yet to be identified.

### 4.2. FGF23 and Outcomes

Serum FGF23 is currently considered to be an early indicator of phosphorus metabolism disorders, and it may be a marker of a renal phosphate homeostasis disorder in patients with early-stage CKD and normal serum phosphate levels. Thus, elevated FGF23 levels are now thought to be a key feature of dysregulated mineral metabolism in patients with CKD [10,96]. Moreover, high FGF23 levels are more strongly associated with mortality, vascular disease, LVH, and kidney disease progression than high serum phosphate levels are [11,100,101,102,103,104].

#### 4.2.1. Chronic Kidney Disease and Dialyzed Patients

a. FGF23 levels and mortality

The association between serum phosphate and mortality among patients with CKD is well established [9,37,39] and has prompted many researchers to examine the relationship between FGF23 levels and mortality. Both phosphate and FGF-23 levels above the normal range have been linked to an increased risk of mortality in non-transplanted patients with CKD [23,24,91]. This association has also been evaluated among patients on dialysis. Gutierrez et al. [100] reported an increased risk of mortality among adult patients on dialysis, and especially among those with the highest FGF23 levels when dialysis was initiated. Similar results were described by Jean et al. [105] where the higher quartiles of serum FGF23 levels two years after inclusion were associated with an increased risk of mortality.

In 2011, Kendrick et al. reported results for 1099 patients with advanced CKD having participated in The Homocysteine in Kidney and End Stage Renal Disease study. After a median (range) follow-up period of 2.9 (2.1–3.7) years, 453 deaths were recorded. Higher serum concentrations of FGF23 were associated with an increased risk of all-cause mortality (HR [95% CI] = 1.63 [1.29–2.07] per SD increment in the log FGF23 concentration, in the fully adjusted model *p* < 0.0001) [106].

b. FGF23 levels and cardiovascular events

An elevated FGF23 level is now considered to be a robust predictor of cardiovascular disease [79] and cardiovascular death [11,100] in patients with CKD. In the study mentioned above, Kendrick et al. assessed the association between FGF23 levels and cardiovascular events. They recorded 215 cardiovascular disease events over a median follow-up period of 2.9 years. Each SD increase in the log serum FGF23 level was associated with a 54% higher risk of cardiovascular events in the fully adjusted models (HR [95% CI] = 1.54 [1.07–2.21]; *p <* 0.02) [106].

Moreover, many researchers have sought to evaluate a putative causal link between FGF23 and LVH. Indeed, clinical observations have revealed associations between high FGF23 levels and the prevalence of LVH [102] or the risk of new-onset LVH [80]. In a prospective cohort of 3860 participants with stage 2 to 4 CKD, Scialla et al. reported that higher FGF23 levels were independently associated with an increased risk of cardiovascular events in general and congestive heart failure in particular. In unadjusted Cox models, the HR [95% CI] for hospitalization for congestive heart failure (comparing the highest FGF23 quartile with the lowest FGF23 quartile) was 8.18 [5.47‒12.24], and the association remained statistically significant after adjustment in many different multivariable models. Scialla et al.’s results also suggest that higher FGF23 levels might be more closely associated with cardiovascular volume overload complications (i.e., LVH and congestive heart failure) than with ischemic heart disease [79]. Hsu et al. had previously described an independent association between FGF23 levels and presence of LVH, which is a predictor of poor cardiac outcomes among chronic hemodialysis patients [107,108]. However, the researchers did not find an association between FGF23 levels and cardiovascular-disease–related death over a median follow-up period of 22 months [109].

#### 4.2.2. Kidney Transplant Recipients

a. FGF23 levels and mortality

The results in cohorts of kidney transplant recipients are in line with the literature data reported in a pre-transplant CKD setting. In a single-center, prospective cohort of 593 stable kidney transplant recipients, Baia et al. [82] recorded 128 deaths over a median (range) follow-up period of 7.0 (6.2–7.5) years. They reported that FGF23 was independently associated with all-cause mortality (full model HR [95% CI] = 1.86 [1.27–2.73]; *p* = 0.001). Similar results were reported earlier by Wolf et al. [76] in a study of a prospective cohort of 984 stable kidney transplant recipients.

b. FGF23 levels and cardiovascular events

Similar results were described concerning the association between high serum levels of FGF23 and an increased risk of cardiovascular mortality [82] in kidney transplant recipients. In the above-mentioned study, Baia et al. [82] also evaluated the association between FGF23 levels and cardiovascular mortality. They found that elevated FGF23 was associated with an higher risk of cardiovascular mortality (in a fully adjusted multivariate Cox regression model: HR [95% CI] = 1.88 [1.11‒3.19]; *p* = 0.02). Moreover, as noted in non-transplanted patients with CKD, higher FGF23 levels might be more consistently associated with cardiovascular volume overload complications (i.e., LVH and congestive heart failure) [82,110] than with ischemic heart disease or atherosclerosis [111,112]. Malyszko et al. [110] reported that both FGF23 and Klotho were related to markers of endothelial cell injury in kidney transplant recipients. They concluded that disturbances in the FGF23-Klotho system were related to endothelial cell injury. Taken as a whole, these results suggest that high FGF-23 levels contribute to the increased cardiovascular risk in kidney transplant recipients by impairing endothelial function. Moreover, the post-transplantation decline in FGF23 levels is paralleled by an improvement in endothelial function. For example, Yilmaz et al. [113] reported that endothelium-dependent vasodilatation increased after kidney transplantation, in parallel with the decrease in FGF23, the reduction in serum phosphorus and the increase in 25-hydroxyvitamin D levels. Although Yilmaz et al.’s study could not prove the causal nature of this relationship, one can hypothesize that part of the post-transplantation decrease in the cardiovascular risk is due to partial resolution of the CKD mineral and bone disorder [113].

## 5. Protein-Bound Uremic Toxins

### 5.1. Serum Levels of Protein-Bound Uremic Toxins

The most extensively studied protein-bound uremic toxins are PCS and IS, both of which are generated in the intestinal tract [114]. Their accumulation in the uremic organism has a negative impact on many physiologic functions including cardiovascular, bone, and renal toxicities [115].

#### 5.1.1. Chronic Kidney Disease and Dialyzed Patients

The serum levels of PCS and IS levels are inversely related to renal function, and free and total concentrations increased progressively with the CKD stage [116,117]. However, in a cross-sectional study including predialysis patients, Eloot et al. [118] have demonstrated that several currently used formulas for calculating the eGFR (and thus the severity of CKD) poorly reflect the accumulation of this uremic retention solute.

Protein-bound uremic toxins are difficult to remove by dialysis. In patients at different CKD stages, we and others have shown that serum levels of total and free PCS and total and free IS were significantly higher in later CKD stages (5 and 5D) [116,117,119]. On the same lines, the serum concentrations of PCS and IS rise as residual renal function falls in incident PD patients [120].

#### 5.1.2. Kidney Transplant Recipients

Only a few reports have evaluated levels of protein-bound uremic toxins in transplanted patients [88,121,122]. In two prospective studies of transplanted patients, serum levels of PCS and IS decreased significantly following transplantation [88,122]. Interestingly, the post-transplantation PCS and IS levels were also markedly lower than in a non-transplant population with identical GFRs at several time points during the study period. There are several possible explanations for this difference: the administration of immunosuppressants, antibiotics or other drugs, or the transplantation procedure itself [123]. It has been suggested that the composition of the colonic microbiota changes after kidney transplantation [124]. All kidney transplant patients are given immunosuppressants and prophylactic antibiotics. A growing body of evidence indicates that antibiotic treatment shifts the position of the colonic microbiota and may therefore affect microbial metabolism after the patient has undergone kidney transplantation [125].

### 5.2. Protein-Bound Uremic Toxins and Outcomes

Protein-bound uremic toxins might constitute the missing link between CKD and a high incidence of CV complications. Data from preclinical studies suggest that uremic toxins can be involved in the pathogenesis of vascular disease [115,122].

#### 5.2.1. Chronic Kidney Disease and Dialyzed Patients

In a study of 139 patients at different CKD stages, we have observed a weak association between serum unbound and total PCS levels and vascular calcification; in contrast, there was no relationship with arterial stiffness as evaluated by measuring the pulse wave velocity [117]. However, serum unbound and total IS levels correlated with not only aortic calcification but also vascular stiffness [117].

In predialysis patients, the serum unbound PCS concentration had been linked to cardiovascular events in a cohort of 499 patients with mild-to-moderate CKD [126]. It should be noted that the association was independent of the GFR and the Framingham risk. Indeed, the main problem in analyzing the link between uremic toxins and outcomes in predialysis patients is how baseline renal function is considered in the statistical models. Other researchers found that high serum PCS and IS levels were associated with the all-cause mortality risk in a group of 268 predialysis patients [127]. On the same lines, our study of a group of patients at different CKD stages showed that the that serum unbound PCS level (but not the total PCS level) was associated with overall and cardiovascular mortality risk independently of other risk factors [116,117].

The links between high PCS and IS levels and cardiovascular events and mortality were confirmed in studies of dialyzed patients [116,128]. In a report on 394 participants in a prospective nationwide American cohort study of incident dialysis patients, a higher PCS level was associated with a greater risk of cardiovascular mortality (HR [95% CI] per SD increment = 1.62 [1.17–2.25]; *p* = 0.004) and a first cardiovascular event (1.60 [1.23–2.08]; *p* < 0.001) in fully adjusted models. Patients in the highest quintile of the combined solute index (incorporating PCS, IS, hippurate, and phenylacetylglutamine) had a 96% higher risk of cardiovascular mortality and a 62% higher risk of a first cardiovascular event, relative to those in the lowest quintile [129].

#### 5.2.2. Kidney Transplant Recipients

There are relatively few data on the associations between protein-bound uremic toxins and hard clinical outcomes in transplanted patients with CKD. Only one report has focused on the relationship between IS levels and cardiovascular events, graft survival, and mortality in a cohort of 311 kidney transplant patients [122]. The IS levels at time of transplantation or one or 12 months after transplantation were not predictive of hard outcomes such as overall mortality, cardiovascular events, and renal endpoints.

## 6. Conclusions

Non-transplanted and transplanted patients with CKD may differ in terms of mortality and the risk of clinical events. This difference is probably due to both traditional and non-traditional risk factors. The levels of the uremic toxins reviewed here and these toxins’ associations with hard clinical outcomes are not always the same in non-transplanted and transplanted patients. To note the renal function could be an important cofounder when the association between uremic toxins and hard outcomes is studied.

## Figures and Tables

**Table 1:** Evolution over time of uremic toxins during chronic kidney disease (CKD) and after renal transplantation.

	Early CKD Stages	Late CKD Stages	Early Transplant (<1 Month)	Early Transplant (<1 Year)	Late Transplant
**Low molecular weight uremic toxins**					
Phosphate	→	↗	↘↘	↘	→
TMAO	→	↗	↘→	→	→
**Middle molecular uremic toxin**					
FGF23	↗	↗↗↗	↗↗↗	↗↗	↗
**Protein bound uremic toxins**					
P cresyl sulfate	↗	↗↗	↘→	→	→
Indoxyl sulfate	↗	↗↗	↘→	→	→

Abbreviations: FGF23: fibroblast growth factor 23; TMAO: Trimethylamine-N-oxide. The direction of the arrows describes the rate of evolution according to the values observed in normal subjects.
